# Type 2 diabetes mellitus: A risk factor for *Helicobacter pylori* infection: A hospital based case-control study

**DOI:** 10.4103/0973-3930.60008

**Published:** 2010

**Authors:** Bikha Ram Devrajani, Syed Zulfiquar Ali Shah, Aftab Ahmed Soomro, Tarachand Devrajani

**Affiliations:** Department of Medicine, Liaquat University of Medical and Health Sciences Jamshoro/Hyderabad (LUMHS), Pakistan; 1 Department of Medicine, Liaquat University Hospital, Hyderabad, Pakistan

**Keywords:** Diabetes mellitus, *Helicobacter* pylori, *Helicobacter pylori* stool antigen

## Abstract

**Objective:**

To determine the frequency of *Helicobacter pylori* (*H. pylori*) infection in diabetic and non-diabetic patients and to compare the frequency of *H. pylori* infection in both groups.

**Study Design:**

Case control.

**Place and Duration:**

Department of Medicine, Liaquat University Hospital from October 2007 to March 2008.

**Materials and Methods:**

This hospital-based case-control study was conducted on 148 subjects and divided into two groups i.e. type 2 diabetics and non-diabetics; each group consisting of 74 patients. All diabetic patients of ≥ 35 years of age, both gender and the known cases with history of dyspepsia, epigastric pain or bloating for more than a month were screened for *Helicobacter pylori* infection. The collected data of both groups was evaluated and separated for analysis.

**Results:**

Majority of the patients were male with mean age ± SD, 52.86 ± 8.51. Among the diabetic group, HpSA was positive in 54/74 (73%), whereas in the non-diabetic group HpSA was positive in 38/74 (51.4%) cases. Fasting blood glucose was identified as low in 04 (5.40%) *H. pylori* infected - diabetic patients where as the blood glucose level of 07 (9.45%) known diabetic patients was raised despite the ongoing medication.

**Conclusion:**

Diabetic patients are more prone and at risk to acquire *H. Pylori* infection. Therefore proper monitoring of blood glucose level and screening for *H. pylori* infection are effective preventive measures for this life threatening infection.

## Introduction

Infection with *Helicobacter pylori* has been recognized as a public health problem worldwide[1] affecting approximately 50% of the world population and more prevalent in developing than the developed countries.[2] It is a common infection in diabetic patients who have inadequate metabolic control as such individuals are colonized by *H. pylori* infection in the gastric antrum, probably because of chemotactic factors such as tumor necrotic factor (TNF), interleukins-IL1, IL2, and IL8 are present in gastric epithelium. These cytokines induce a number of changes in the gastric epithelium that promote inflammation and epithelial damage thus leading to increased risk of aberrant repair giving the picture of gastric atrophy or epithelial cell metaplasia.

Diabetes mellitus is one of the important causes of dyspepsia. Disordered gastrointestinal motor function is now recognized as a major cause of diabetes mellitus. Beside DM the *H. pylori* is also a well established cause of dyspepsia. The incidence of *Helicobacter pylori* is increased in diabetes mellitus.[3] Delayed gastric emptying and antral dysmotility are important causes of dyspepsia in diabetes. The role of *Helicobacter pylori* infection in diabetic dyspepsia is mainly related to blood glucose concentration. Hyperglycemia may induce the infection by *H. pylori* or the silent infection may get reactivated and produce symptoms of dyspepsia in diabetes.

The prevalence of diabetes mellitus in Pakistan is 22%,[4] the prevalence of *Helicobacter pylori* is 49%[5] whereas the prevalence of *Helicobacter pylori* in diabetes mellitus is 61%.[6]

Diabetes is diagnosed according to the diagnostic criteria for the diabetes mellitus[7] whereas the diagnostic tools for *Helicobacter pylori* infection are serology, rapid urease test (RUT), urea breath test (UBT), endoscopy and biopsy/histopathology, polymerease chain reaction (PCR) for DNA of *H. pylori* and *Helicobacter pylori* stool antigen (HpSA).[8] The simplest test of *Helicobacter pylori* is serologic, including the assessment of specific IgG levels in serum but it cannot be used for early follow-up and has high rates of false positive results.[9] The urea breath test is non-invasive but the radioactive isotope^14^C exposes the patient to radiation. Another more specific, rapid and newly researched non invasive test is *Helicobacter pylori* stool antigen (HpSA). The premier platinum HpSA enzyme immunoassay (EIA) is an *in vitro* qualitative procedure for the detection of *Helicobacter pylori* antigen in human stool.[10] It can be performed in 90 minutes with an overall specificity and sensitivity of 94% by doing HpSA.

Hyperglycemia is controlled by insulin or oral hypoglycemic agents while the drugs used for eradication of *Helicobacter pylori* infection are proton pump inhibitors, bismuth compounds, metronidazole, clarithromycin, amoxicillin and tetracycline.[11]

Since there are only a few studies in our country on the association of *Helicobacter pylori* and diabetes mellitus, we conducted this study at a tertiary care teaching hospital of Hyderabad, Sindh Pakistan. The study focus is on the frequency of *Helicobacter pylori* infection in patients with type 2 diabetes mellitus and help in providing data that is useful in the field of medicine as well as epidemiology.

## Materials and Methods

This case-control study was carried out in the department of Medicine at Liaquat University Hospital (a tertiary care 1500 bedded hospital) Hyderabad, Pakistan from October 2007 to March 2008.

The inclusion criteria of study were: All patients (1) above 35 years of age, (2) either gender, (3) with history of dyspepsia, bloating or epigastric discomfort for more than one month, through outdoor patient department (OPD), (4) who were known cases of type 2 diabetes mellitus of approximately five years duration and came with history of dyspepsia, epigastric discomfort, or bloating for ≥30 days.

The exclusion criteria of study were: (1) Patients of type-1 diabetes (2) Non-cooperative patients who refuse to give consent or participate in the study (3) Patients already on steroid or immunosuppressive or *H. pylori* eradication therapy.

The inclusion criteria of study were then investigated for diabetes (if not known) and *Helicobacter pylori* infection, and divided into two groups - A and B. Group A (also labeled diabetic group) contains patients of diabetes mellitus (newly diagnosed or known cases) with positive or negative *Helicobacter pylori* infection while group B (also labeled non diabetic group) contains non diabetic individuals with positive or negative *Helicobacter pylori* infection. Each group (A and B) consists of 74 patients. The known cases of diabetes mellitus in group A were also investigated for blood sugar (not for diagnostic purpose but to assess the blood sugar level that whether it is controlled or uncontrolled).

### Data Collection Procedure

1. For the assessment of diabetes mellitus we had taken venous blood sample and send to laboratory for fasting blood sugar (FBS) level, random blood sugar (RBS) level and hemoglobinA1c
2. For detection of *Helicobacter pylori* infection we advised the participants for collection of stool sample and send to laboratory for *Helicobacter pylori* stool antigen (HpSA) by Enzyme immunoassay (EIA).

The collected data of both groups (A and B) were then evaluated, separated and saved for analysis.

### Data analysis

The data were evaluated in statistical program SPSS version 11.0. Frequency and percentages were calculated on 95% confidence interval. Mean ± SD of age was computed among the numerical parameters. The Chi-Square test was applied among the categorical variables. The *P* value < 0.05 was considered as statistically significant.

## Result

**Figure 1:**
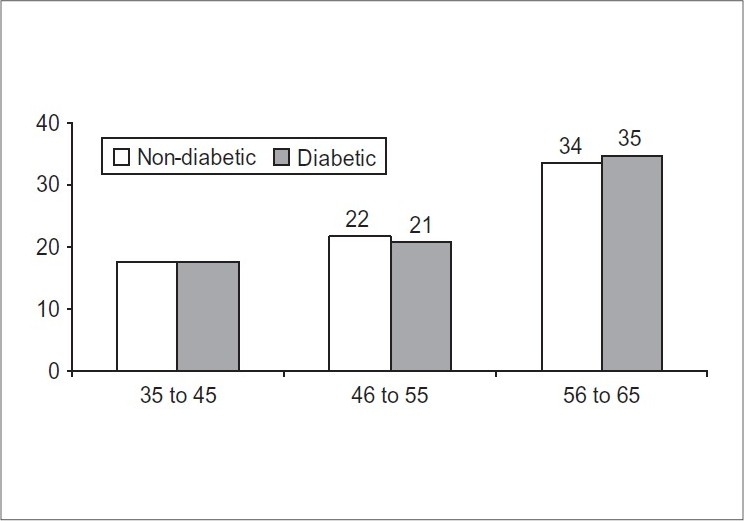
Evaluation of cases within diabetic and non-diabetic group A (Diabetic) and B (Non diabetic group); (N = 148)

**Figure 2:**
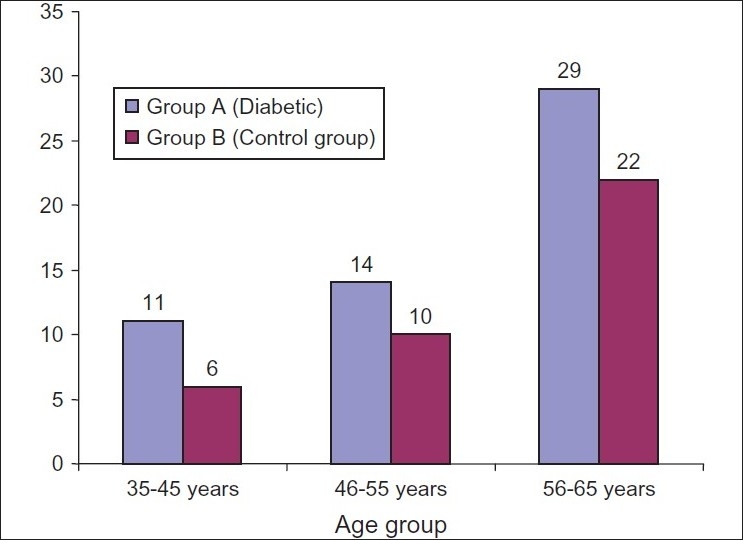
Frequency of *Helicobacter pylori* infected patients in relation to age and group

Seventy four patients in each group were studied, of which 83 (56%) were males and 65 (44%) females, mean age 1 SD 52.86 1 8.51. Majority of the patients were more than 50 years of age [Table 1]. The frequency of patients in relation to age group is mentioned in [Figure 1]. In the diabetic group, HpSA was positive in 54/74 (73%) cases while in non-diabetics, HpSA was positive in 38/74 (51.4%) cases [Table 2]. Majority of the *H. pylori* infected patients in both groups were more than 50 years of age [Table 3]. The *H. pylori* infection in relation to frequency and age in both diabetic and non diabetic group is mentioned in [Figure 2]. In the diabetic group, out of 74 cases, 52 (70%) belonged to urban areas while 22 (30%) belonged to rural areas; in the non diabetic group, out of 74 cases, 49 (66%) belonged to urban areas and 25 (34%) belonged to rural areas. In both groups the *Helicobacter pylori* infection was more predominant in patients belonged to urban areas. In the diabetic group, 10 patients withheld or withdrew their diabetic treatment but despite discontinuation of medicine the fasting blood glucose level was low.

**Table 1:** Demographic distribution of patients (N = 148)

Age (in years), Mean ± SD (Range)	52.8 ± 8.51 (35-65)
Gender	N (%)
Male	83 (56.1)
Female	65 (43.9)
HpSA results	
Positive	92 (62.2)
Negative	56 (37.8)
Age group	
35 to 45	36 (24.3)
46 to 55	43 (29.1)
56 to 65	69 (46.6)

**Table 2:** Frequency of *Helicobacter pylori* stool antigen positive cases in diabetic and non diabetic group

Parameter	HpSA (n = 148)		*P* value
		
	Non-diabetic (N = 74)%	Diabetic (N = 74)%	
HpSA			
Positive	38(51.4)	54(73.0)	0.0001†
Negative	36(48.6)	20(27.0)	

† *P* value is statistically highly significant

**Table 3:** Frequency of *Helicobacter pylori* stool antigen positive cases in relation to age

Parameter	HpSA (*n* = 148)		*P* value
		
	Negative (N = 56)%	Positive (N = 92)%	
Age group			
35 to 45	19 (33.9)	17 (18.5)	0.01
46 to 55	19 (33.9)	24 (26.1)	
56 to 65	18 (32.1)	51 (55.4)	

## Discussion

Patients with diabetes mellitus are often affected by chronic infections. Many studies have evaluated the prevalence of *H. pylori* infection in diabetic patients and the possible role of this condition in their metabolic control. Some studies found a higher prevalence of the infection in diabetic patients and reduced glycemic control while others did not support any correlation between metabolic control and *H. pylori* infection.[12]

The present study determined the relationship between type 2 diabetes mellitus and *Helicobacter pylori* infection and found that diabetic patients are more prone to acquire *H. pylori* infection (*P* = 0.0001) (statistically significant); the similar results were also detected in the study conducted at Japan by Kimiaki *et al*.[13] However, the higher prevalence of *H. pylori* infection was also reported in diabetes mellitus than in non-diabetics in a study by Marrollo.[14]

In our study majority of the patients with *H. pylori* infection in both groups - diabetic and non diabetic, were more than 50 years of age where as in another study the mean age was 60 years.[15] Similarly a study conducted at Abakaliki by Ugwu had shown that majority of *Helicobacter pylori* infected patients were more than 60 years of age.[16] However, a study by Sargýn *et al*. shows that the mean age of diabetic patients with *H. pylori* infection is 56 years.[17]

Most of the *Helicobacter pylori* related diseases are associated with male gender, the role of gender as a risk factor for *H. pylori* infection is still debated. The present study shows that the *Helicobacter pylori* infections were more common among males while another study conducted by Catherine confirms the male predominance of *H. pylori* infection in adults as a global and homogeneous phenomenon.[18] On the other hand, in another study the *Helicobacter pylori* infected females were predominant as compared to males,[19] and that contradicts our statement.

In our study, while the selection of patients was made according to the dyspeptic symptoms the lack of statistically significant difference in dyspeptic symptoms between diabetics and non-diabetics corroborates the findings by Anatesios *et al*.[20]

*H. pylori* infection and *H. pylori* related gastrointestinal/gastroduodenal disorder may be related to glycemic status. In our study, the 10 *Helicobacter pylori* infected diabetic patients were detected to have low fasting blood glucose level. However, this is in contrast with the finding of KO *et al*.[21] Peach and Barnnet[22] had previously shown that women infected with *H. pylori* had lower mean fasting plasma glucose concentration than did non-infected women. The lower fasting plasma glucose in *H. pylori*-infected than non-infected diabetics may partly be attributed to alteration in gastric mucosa as high prevalence of severe acute gastritic inflammation/ulcer disease has been reported in diabetic patients with little or no symptoms of dyspepsia.[23] *H. pylori* gastritis has been found to enhance glucose and meal stimulated insulin release probably by increasing gastrin secretion.[24] However, no association has yet been documented between *H. pylori* infection and delayed gastric emptying or upper gastrointestinal symptoms in diabetics.[25]

*H. pylori* may have been acquired earlier in life independent of glycemic status and prior to the development of type 2 diabetes mellitus and subsequently confers some degree of protection against excessive elevation of blood glucose. Wu *et al*.[26] have hypothesized that lack of *H. pylori* infection, especially during childhood, might enhance the risk of development of morbid obesity (a known risk factor for diabetes mellitus) based on their finding of inverse relationship between morbid obesity and *H. pylori* infection.

Regarding the diagnostic tool used in our study for the detection of *Helicobacter pylori* infection, we preferred and use *Helicobacter pylori* stool antigen test (HpSA) because it is rapid and noninvasive method with high sensitivity (94%) and specificity (94%) and is potentially very helpful in diagnosing active and repeated *H. pylori* infection.[27] In addition the test may be used within days of the initiation of anti *H. pylori* therapy to confirm efficacy and assess patient compliance. Successful eradication can be confirmed with a negative result at least four weeks following completion of therapy. Therefore, keeping such studies in mind it appears that stool test can be used as a reliable maker for initial screening of *H. pylori* infection.[28] In our study the majority of patients with *Helicobacter pylori* infection were between 56-65 years of age and this is similar to the study by Zhang.[29]

Regarding the demographical presentation of current study, majority of the diabetic patients with *Helicobacter pylori* infection belong to the urban areas of province Sindh Pakistan and this finding resembles with the study conducted at Benin during 2003-2004 in which 75.4% of peoples were belong to urban population;[30] a similar finding was also detected in the study of Hoang published in the year 2005.[31]

The present study detects that hyperglycemia is a risk factor for *Helicobacter pylori* infection. The Hisayama study by, Yamagata *et al*. shows that hyperglycemia is a possible cofactor increasing the risk posed by *H. pylori* infection.[32]

## Conclusion

Overall, the present study suggests that diabetic patients are at more risk for *H. Pylori* infection in comparison to non-diabetic population so every diabetic patient with acid peptic disorder must be screened for *H. Pylori*. There is a dire need to provide proper counseling, education and awareness regarding diabetes mellitus and its association with *H. pylori* infection. Effective and appropriate measures should be taken against control of diabetes mellitus, eradication of *H. pylori* infection.
